# The Effect of Twin Sex on Menstrual Characteristics

**DOI:** 10.3390/medicina56040173

**Published:** 2020-04-10

**Authors:** Elizabeth Kowynia, Shayesteh Jahanfar

**Affiliations:** Department of Public Health, Central Michigan University, Mt Pleasant MI 48859, USA; jahan2s@cmich.edu

**Keywords:** menstruation, period, heavy, twin, sex, hormones

## Abstract

*Background and objectives*: The purpose of this project is to evaluate the association between twin sex discordance and menstrual characteristics. We hypothesize that sharing the uterus with a male twin can change ovulation programming, hence changing the menstrual cycle characteristics during adulthood. This project could be novel in discovering new physiological mechanisms of hormone exposure and menstrual cycles. *Materials and methods*: This is a cross-sectional study. We asked females from sex-concordant (*n* = 1290) and sex-discordant (*n* = 168) twin pairs in the Washington State Twin Registry about characteristics of menstrual cycles. Generalized Estimating Equation (GEE) analysis was used to compare groups. The main outcome measures included the amount of bleeding, duration of menstruation, the timing of menstruation, length of menstruation, and a number of periods per year. *Results*: We found a statistically significant association between the amount of menstrual period bleeding and twin sex discordance (0.42 (95% CI 0.18–0.94)). However, twin sex discordance was not associated with period duration, length of menstrual cycle, cycle regularity, or a number of periods per year. *Conclusions*: Twin sex discordance is not a predictor of clinical characteristics of menstruation during adulthood except for the amount of bleeding. Future studies should focus on the impact of male hormones on the amount of bleeding during menstruation.

## 1. Introduction

The literature surrounding the impact of fetal sex discordance and its effects in menstrual characteristics is scarce. Animal studies have shown an association between sex discordance and general psychological and physiological characteristics. In female mice, it is possible to predict genital morphology, the timing of puberty, length of estrous cycles, and timing of menopause based on the mouse’s position in the womb to other male and female mice [1]. Female rodents in between two male rodents in utero have higher instances of trait masculinization [2]. Studies in humans have shown a potential for male hormonal transfer from male to female twins in utero. There is some evidence to support that females with a male twin have a lower second to fourth finger ratio than that of females with a female twin, suggesting a higher exposure to testosterone in the womb [3]. However, these data were not always consistent when replicated [4]. Female infants also show an increased ability to rotate an image of an object in one’s mind when they have a male twin, which is a masculine trait in infants [5]. In 2018, Jahanfar and colleagues investigated the effect of sex discordance on placenta pathology. Female twins from sex-discordant pairs were less likely to have placental anastomosis, which causes twin-to-twin transfusion syndrome, compared to the twins of the same sex. Males from male-male pairs had higher odds of unequal placental sharing (74% higher) and composite inflammation (52% higher) compared with the males from sex-discordant twins [6]. Female infants with a male twin have also been shown to use areas of the brain more common for male infants to use compared to their female-female pair counterparts [7]. Controversy among the current literature supports the need for further research on this topic. We were unable to find any research about the impact of twin sex discordance on menstrual characteristics.

Studies have indicated that females who share a uterus with males have differences in reproductive traits. Fetal rabbits treated with testosterone have significantly longer anogenital distances and more chin marks (masculinized traits), as well as higher weights, more aggression, higher blood testosterone as adults, and later vaginal opening when compared to their unexposed counterparts [8]. In humans, higher fasting serum levels of sex hormone-binding globulin and free androgen indices as adults are associated with increased risk of prolonged and abnormal menstrual cycles, independent of obesity [9]. Menstrual irregularity is also shown to be influenced more by environmental factors rather than genetic factors when dizygotic twins were compared [10]. Based on current research, exposure to testosterone in the womb from a sex-discordant twin has the potential to influence menstrual irregularity in adulthood, but more research is necessary. No studies have compared menstrual characteristics between sex-concordant and sex-discordant twins, which is why this study is novel.

In humans, it is shown that females who have sex-discordant twins have higher body weights than their sex-concordant counterparts, even though their birth weights do not significantly differ [11]. Higher weights are associated with hyperandrogenism and increased instances of menstrual abnormalities, diagnosed as a polycystic ovarian syndrome in humans [12]. Females with sex-discordant twins also have significantly higher instances of hyperlipidemia compared to females with sex-concordant twins [11]. The exchange of steroid hormones between twins in the womb could be a potential explanation for these phenomena. The current project is novel because it could lead to more answers regarding menstrual irregularity. This study will investigate the correlation between male twin exposure and menstrual characteristics in humans. If the current project could apply the concept of steroid hormone exchange between human twins, it would help to paint a picture of how the human body and its physiological processes function.

More research on this topic is necessary to investigate if menstrual abnormalities are associated with the presence of a sex-discordant twin. The current hypothesis is that sharing a fetus with a male twin has an effect on menstruation during adulthood. If it does, this could further support the hypothesis that sex-discordant twins can exchange steroid hormones to each other in utero, and the testosterone exposure affects the reproductive physiology of the female twin.

## 2. Materials and Methods

Participants recruited in this study included 1458 female individual twins (994 same-sex monozygotic twins (SSMZ), 296 same-sex dizygotic (SSDZ) and 168 opposite-sex dizygotic twins (OSDZ)) from the Washington State Twin Registry (WSTR). The WSTR (www.wstwinregistry.org) has over 9000 twin adult sets in various ages (18–70 years old), sex (female/female pairs 50.2%, male/male pairs 27.9% and male/female pairs 21.9%), and zygosity (identical 51.9%, non-identical 48.0%). The current study used the WSTR to collect data on reproductive health among female twins. All females with a female or male twin were included in this study. This is a cross-sectional study. This study was approved by the Washington State University IRB number 14515. WSTR IRB approval (Reference number: R17-03) agreement was signed by PI on 21 February 2017.

Participants included were females with either a male or female twin who were registered through the WSTR and responded to the survey. Participants were excluded within each test variable if they failed to respond to its corresponding question(s).

The sample size was calculated using power at 80 and the Type I Error rate at *α* = 0.05, given the frequency of menstrual characteristics from Jahanfar [10]. We chose the largest sample calculation and added 20% to the final sample size.

The survey was sent electronically, so the twins approached include those with a valid email address. The survey was built using an online platform and pairs in which both members of the pair completed the study has entered a raffle for a $10 gift card. Of the over 18,000 twins in the registry, 9110 individual twins were approached. This study had a response rate of 22.1%. The participants were sent a researcher’s statement, and consent and contact information were provided for further inquiries and questions. The survey was sent by email in three rounds.

This survey was sent to all registered twins in WSTR with little incentive to respond, which may explain the low response rate. It is also worth noting that respondents were highly educated, suggesting that respondents may have had more education than non-respondents. Given the sample age, it is also possible that respondents were older than non-respondents. All valid responses were analyzed within each category.

The participants answered questions regarding reproductive health. This includes, but is not limited to, menstrual duration, menstrual cycle length, menstrual regularity, menstrual period amount, and the number of periods experienced in a typical year. Post-menopausal women were asked to recall from reproductive age. Using this data, female twins were divided into two groups based on the sex of their twin: the sex-concordant group and the sex-discordant group. The twin pairs were coded as F.F. (female-female twin pair) = 1 and F.M. (female-male) twin pair = 2. The five variables compared between these two groups are defined below.

Menstrual period duration: Menstrual period duration is defined as the number of days a person bleeds during their menstrual period. Participants were asked, “about how many days does your period usually last?” The range of available responses to the questionnaire spanned from two days to eight days or more.

Menstrual cycle length: Menstrual cycle length is defined as the number of days that occur between menstrual periods. This was calculated by counting the number of days between the first day of the menstrual cycle from the previous cycle till the first day of menstruation of the current cycle. Participants were asked, “on average, how long is your cycle? Your cycle is the length of time between your periods.” The range of available responses to the questionnaire was 21 days to 35 days or more. These responses were coded as a range of 21–35 days for data analysis.

Menstrual regularity: Menstrual regularity is defined as the degree of consistency in menstrual duration and cycle length. Participants were asked, “Are your periods regular, that is, do they usually start within a day or two of when you expect them to?” Participants could either answer yes or no. These answers were coded as yes = 1 and no = 0.

Menstrual period amount: Menstrual period amount is defined as the amount of endometrial shedding that occurs during a menstrual period. Participants were asked, “how would you describe your bleeding?” Participants could answer “light,” “normal,” or “heavy.” Light was defined to participants as “spotting or changing one pad per day during your period.” Normal bleeding was defined as “having a flow of blood or changing 2 to 3 pads per day during your period.” Heavy bleeding was defined as “excessive bleeding or having to change more than 3 pads per day during your period.” For the purposes of this study, light and regular amounts were grouped together. These variables were coded as light + normal = 1 and heavy = 2.

Menstrual period frequency: Menstrual period frequency is defined as the number of times per year a participant recalls having their period. Participants were asked, “about how many times per year do you have your period?” Available responses ranged from 1 period to 24 periods in a year.

Several potential confounders were adjusted for in the analysis of this data. These demographic variables include race, education, ethnicity, income, college enrollment, employment, relationship status, and age. These demographic factors were included to account for various stressors that could have altered menstruation. All responses from eligible participants were included in this study. The study is limited in that participants only reside in Washington State, but all members of the WSTR were sent the survey, which is inclusive of the entire state. Because all eligible responses were used, a study size of 1458 female twins was reached. Additionally, same-sex twin pairs were not differentiated by zygosity. All monozygotic twin pairs were included in the sex-concordant twin group.

Descriptive and bivariate analysis were computed: *t*-test was used for continuous variables, and the Chi-square test was used for categorical data comparisons. *p* < 0.05 was considered statistically significant in this study. Cluster regression analysis or Generalized Estimating Equation (GEE) regression analysis was used. GEE analysis was run for each menstrual characteristic; both unadjusted and adjusted risk was estimated. Odds ratio (OR) was used for each categorical variable, while the risk ratio (R.R.) was used for each continuous variable. For each subcategory, missing responses were excluded from the data analysis.

Twins provide already matched pairs or clusters within-twin-pair and between-twin-pair effects. Because of this, a specialized standard regression model that reflects the paired structure of the data should be used, which manifests the correlation between twins. The statistical analysis included estimating several factors influencing menstrual characteristics between groups using GEE modelling for bivariate and multivariate analysis. Statistical analyses were performed using IBM SPSS Statistics for Windows, version 24 (IBM Corp., Armonk, NY, USA).

## 3. Results

The sample included 1458 individual female twins (age 19.24–71.38) who were both eligible and responded to the survey. All responses from eligible participants were included, and only missing data were excluded at each stage. Of this sample, participants were 91.4% white, 8.7% non-white, and 3.5% Hispanic. The majority of the sample holds a bachelor’s degree or less, make less than $79,999 per year, are single, employed, not currently a student, and have a mean age of 45.45 ± 14.15 years (Table 1). Menopause was not assumed based on age and data marked not applicable by the participant was excluded at each stage. In terms of menstrual characteristics, 74.3% had light-average period amounts, 68.4% had regular periods, had an average cycle length of 28.12 days, an average period duration of 5.05 days, and an average menstrual frequency of 8.73 periods per year (Table 1). The majority of the sample came from a sex-concordant pair, with 88.5% from a female-female pair (Table 1).

In this study, there were no statistically significant differences in race, education, ethnicity, income, college enrollment, employment, relationship status, menstrual period regularity, or period amount between females from sex-concordant and sex-discordant twin pairs when a Chi-squared test was performed (Table 2). There was also no significant difference among sex-concordant and sex-discordant groups in age, menstrual cycle length, period duration, or menstruation frequency when an independent sample t-test was performed (Table 2). Bivariate analysis was performed using GEE analysis, as shown in Table 3. The results of which are listed below.

### 3.1. Amount of Menstruation

The unadjusted odds ratio showed that the amount of menstruation and twin sex concordance is not statistically significantly associated (0.64 (0.37–1.10)). However, the adjusted odds ratio showed a statistically significant association between menstrual period amount and twin pair sex (0.42 (0.18–0.94)). The exposure to a male twin reduced the odds of heavy bleeding by 58% (0.42 (0.18–0.94)).

### 3.2. Regularity

The unadjusted odds ratio showed menstrual period regularity, and twin sex concordance is not associated (1.17 (0.73–1.89)). The adjusted odds ratio was also not statistically significant (1.59 (0.83–3.04)).

### 3.3. Menstrual Cycle Length

The unadjusted risk ratio showed no association between menstrual cycle length and twin sex concordance (1.04 (0.43–2.50)). The adjusted risk ratio also was not statistically significant (1.17 (0.42–3.31)).

### 3.4. Period Duration

The unadjusted and adjusted risk ratios showed no associations between period duration and twin sex concordance (0.94 (0.68–1.29)).

### 3.5. Period Frequency

The unadjusted risk ratio showed no association between a number of periods per year and twin sex concordance (0.33 (0.05–2.12)). The adjusted risk ratio was also not statistically significant (0.26 (0.02–3.97)).

## 4. Discussion

Prior to this study, it was hypothesized that twin sex would have an effect on menstrual characteristics in the female twin. This study supported the hypothesis that females with a male twin are 58% less likely to have heavy menstrual periods compared to females with a female twin (0.42 (0.18–0.94)). There are many reasons as to why menstrual amount differs between females with a female twin and females with a male twin. One of which could be the hypothesis that steroid hormones are exchanged between twins in utero, leading to physiological changes after birth. This hypothesis was supported by Cohen-Bendahan and colleagues who found female infants with a male twin have also been shown to have a more masculine cerebral lateralization than their counterparts with a female twin [7] and Van-Anders and colleagues when they found females with a male twin have a lower second to fourth finger ratio than that of females with a female twin, indicating a higher exposure to testosterone in the womb [3]. The transfer of steroid hormones from twins leading to fetal androgen or estrogen excess reprograms ovarian development and reproductive neuroendocrinology, which may lead to menstrual differences later in life [13]. This study is novel in that there are no studies similar to the current one comparing twin sex and menstrual characteristics. This study supports that having a male twin can alter reproductive physiology later in life, possibly explained by the theory that steroid hormones are exchanged between twins in utero.

The current research could have clinical implications in female reproductive health. Females who have a female twin have higher odds of having heavy periods than females with a male twin (Table 3). Women who experience heavy periods have a higher pain score both during menstruation and during transvaginal examinations when compared to a control group [14]. If women with a female twin have a higher risk for heavy periods, they may also be at a higher risk for pain during menstruation, but more research on this topic is necessary. Pain is not the only complication that accompanies heavy periods, however. It is suggested that hospitalization due to iron deficiency anemia in adolescent girls who have heavy menstrual bleeding is an underemphasized problem, and prevention is key [15]. The current study could help identify risk factors for heavy menstrual bleeding and prevent gynecological problems associated with heavy menstrual bleeding. 

In a qualitative study, women who suffer from heavy periods (menorrhagia) describe the condition as affecting their lives physically, emotionally, socially, and financially [16]. In an online survey, heavy bleeding was found to be the most common menstrual disorder among respondents [17]. This publication also claimed undiagnosed bleeding disorders to be a significant health concern due to a lack of knowledge and discussion surrounding abnormal menstrual bleeding. The current study could be used to target at-risk populations for heavy periods so that resources may be effectively distributed in public health initiatives. This information could be used to prevent some of the adverse outcomes of menorrhagia identified above and reduce the lack of education surrounding abnormal menstrual bleeding.

Because this study is novel, there are no replication studies to further support this data. More research should be done among twins to determine if exposure to a male twin in utero affects female reproductive physiology as an adult. Future cohort studies should investigate if females with a male twin have less pain associated with menstrual bleeding. Future studies should also investigate if females with a male twin have fewer instances of hospitalization due to gynecological problems associated with heavy bleeding. They could also examine if twin pair sex is associated with fertility in adulthood. Future studies should also have more samples with more power and less variation. It is possible that if there were more samples, there could or could not have been a statistically significant association between menstrual period regularity and twin sex (Table 3). 

This study had many strengths, the first of which being that it is the first of its kind. This type of novel study will serve as a basis for future research into steroid hormone transfer between twins in utero and its implications on reproductive physiology later in life. The cohort used was large (*n* = 1458) but could have been larger. Future studies could aim to increase the sample size. Additionally, the response rate was low (22.1%), which may have produced a bias in those who responded. The data also came from the Washington State Twin Registry (WSTR), so most subjects resided in Washington state. For data that could be extrapolated to apply to the rest of the United States, a more inclusive cohort should be used. This study was also prone to recall bias, and therefore future studies could use cohorts where subjects are followed rather than asking them to recall information. There were also limitations in that data in that reproductive disorders, or the use of hormonal contraceptives was not accounted for. Factors such as menopausal symptoms, BMI, and subjective stress were not included in this data. Further studies could account for these influences on menstruation. The potential difference in menstruation was also not investigated between sex-discordant dizygotic twins and sex-discordant monozygotic twins. Future studies could investigate these potential differences in menstruation.

## 5. Conclusions

Heavy menstrual periods are an essential issue among women and can lead to serious health consequences. When compared to females with a female twin, females with a male twin are 58% less likely to have heavy menstrual periods. There were no significant differences in period duration, cycle length, period frequency, nor period regularity between females from sex-concordant and females from sex-discordant twin pairs. These results may be due to hormone transfer between twins in utero. However, the impact of twin sex on reproductive physiology needs to be studied further.

## Figures and Tables

**Table 1:** Descriptive analysis of demographic and menstrual variables. Consistent distribution found among females from a sex-concordant pair and females from a sex-discordant pair among all groups.

Categorical Variable	Frequency	Percentage
Race		
- White	1343	91.40%
- Non-white	127	8.70%
Education		
- Bachelor’s or less	1012	70.70%
- Graduate degree	419	29.30%
Ethnicity		
- Hispanic	50	3.50%
- Non-Hispanic	1378	96.50%
Annual Income		
- $79,999 or less	560	61.70%
- $80,000 or more	347	38.30%
College Enrollment		
- Student	108	7.60%
- Non-student	1316	92.40%
Employment Status		
- Employed	1067	74.80%
- Unemployed	360	25.20%
Relationship Status		
- In a relationship	399	27.90%
- Single	1031	72.10%
Twin sex		
- Sex-concordant pair	1290	88.50%
- Sex-discordant pair	168	11.50%
Menstrual Period Amount		
- Heavy	239	25.70%
- Light-Average	690	74.30%
Menstrual Period Regularity		
- Regular	636	68.40%
- Irregular	294	31.60%
**Continuous Variable**	**Mean**	**Standard Deviation**
Age (years)	45.45	14.15
Menstrual Cycle Length (days)	28.12	4.01
Menstruation Frequency (periods/year)	8.73	4.29
Menstrual Period Duration (days)	5.05	1.19

**Table 2:** Menstrual characteristics and demographic group significance between sex-concordant and sex-discordant pairs. An independent sample t-test was used for continuous variables, and the chi-squared test of significance was used for categorical variables. These two tests show no statistical significance in any category between females from a sex-concordant pair and females from a sex-discordant pair.

Categorical Variable	Females from FF Pair (*n* = 1290)		Females from FM Pair (*n* = 168)		*p* Value
Race					0.459
- White	1150	88.80%	145	11.20%	
- Non-white	110	86.60%	17	13.40%	
Education					0.894
- Bachelor’s or less	896	88.50%	116	11.50%	
- Graduate degree	372	88.80%	47	11.20%	
Ethnicity					0.096
- Hispanic	48	96.00%	2	4.00%	
- Non-Hispanic	1218	88.40%	160	11.60%	
Annual Income					0.545
- $79,999 or less	498	88.90%	62	11.10%	
- $80,000 or more	313	90.20%	34	9.80%	
College Enrollment					0.242
- Student	92	85.20%	16	14.80%	
- Non-student	1170	88.90%	146	11.10%	
Employment Status					0.685
- Employed	943	88.40%	124	11.60%	
- Unemployed	321	89.20%	39	10.80%	
Relationship Status					0.97
- In a relationship	354	88.70%	45	11.30%	
- Single	914	88.70%	117	11.30%	
Menstrual Period Amount					0.105
- Heavy	222	92.90%	17	7.10%	
- Light-Average	616	89.30%	74	10.70%	
Menstrual Period Regularity					0.511
- Regular	571	89.80%	65	10.20%	
- Irregular	268	91.20%	26	8.80%	
**Continuous Variable**	**Mean**	**SD**	**Mean**	**SD**	**P**
Age (years)	46.85	14.38	45.98	14.74	0.192
Menstrual Cycle Length (days)	28.13	4.01	28.09	4.05	0.96
Menstruation Frequency (periods/year)	8.63	4.26	9.74	4.45	0.056
Menstrual Period Duration (days)	5.04	1.18	5.11	1.28	0.755

**Table 3:** Generalized Estimating Equation (GEE) analysis of menstrual characteristics. GEE analysis shows a statistically significant difference in menstrual period amount between F.F. and F.M. groups. No significant difference in period duration, cycle length, period frequency, and period regularity were found between groups. The adjusted odds of having a higher amount of menstruation was 58% lower in females from sex-concordant twins compared with sex-discordant twins (95% CI 0.18–0.94).

Amount		
	Unadjusted OR95% CI (Lower-Upper)	Adjusted OR95% CI (Lower-Upper)
Pairsex	0.64(0.37–1.10)	0.42(0.18–0.94)
Race	0.89(0.54–1.45)	0.73(0.37–1.45)
Hispanic/Non-Hispanic	0.87(0.41–1.85)	1.20(0.48–2.98)
Income	1.06(0.73–1.55)	1.03(0.70–1.53)
Age	1.00(0.99–1.01)	1.00(0.99–1.01)
Education	0.83(0.60–1.14)	0.72(0.48–1.06)
Relationship status	0.94(0.69–1.28)	1.10(0.64–1.90)
Employment	1.16(0.78–1.72)	1.01(0.60–1.70)
Student/Non-student	1.15(0.73–1.84)	0.79(0.34–1.84)
**Regularity**		
	**Unadjusted OR** **95% CI (lower-upper)**	**Adjusted OR** **95% CI (lower-upper)**
Pairsex	1.17(0.73–1.89)	1.59(0.83–3.04)
Race	0.94(0.60–1.47)	0.82(0.45–1.49)
Hispanic/Non-Hispanic	1.00(0.50–2.00)	1.13(0.48–2.65)
Income	0.89(0.63–1.27)	0.86(0.60–1.24)
Age	1.00(0.99–1.01)	1.00(0.99–1.01)
Education	1.01(0.76–1.37)	1.08(0.75–1.56)
Relationship status	1.07(0.80–1.43)	1.04(0.61–1.77)
Employment	1.09(0.74–1.60)	1.09(0.68–1.76)
Student/Non-student	0.87(0.56–1.35)	0.70(0.35–1.42)
**Menstrual Cycle Length**		
	**Unadjusted R.R.** **95% CI (lower-upper)**	**Adjusted R.R.** **95% CI (lower-upper)**
Pairsex	1.04 (0.43–2.50)	1.17 (0.42–3.31)
Race	0.75 (0.48–2.81)	1.72 (0.47–6.30)
Hispanic/Non-Hispanic	1.36 (0.32–5.89)	1.56 (0.24–10.00)
Income	1.25 (0.65–2.42)	1.15 (0.59–2.25)
Age	1.01 (0.99–1.03)	1.00 (0.98–1.03)
Education	0.58 (0.33–1.01)	0.60 (0.31–1.16)
Relationship status	1.45 (0.84–2.50)	0.76 (0.28–2.07)
Employment	1.33 (0.67–2.65)	0.94 (0.40–2.22)
Student/Non-student	1.86 (0.78–4.45)	0.75 (0.19–3.00)
**Period duration**		
	**Unadjusted R.R.** **95% CI (lower-upper)**	**Adjusted R.R.** **95% CI (lower-upper)**
Pairsex	0.94 (0.68–1.29)	0.79 (0.51–1.22)
Race	0.92 (0.68–1.24)	0.87 (0.57–1.32)
Hispanic/Non-Hispanic	0.76 (0.47–1.25)	0.76 (0.40–1.44)
Income	1.07 (0.84–1.37)	1.10 (0.86–1.41)
Age	1.00 (1.00–1.01)	1.00 (0.99–1.01)
Education	0.97 (0.80–1.18)	1.04 (0.82–1.32)
Relationship status	1.20 (1.00–1.45)	1.10 (0.76–1.60)
Employment	0.93 (0.73–1.19)	0.94 (0.68–1.30)
Student/Non-student	1.11 (0.84–1.48)	0.94 (0.52–1.71)
**Frequency (Periods per year)**		
	**Unadjusted R.R.** **95% CI (lower-upper)**	**Adjusted R.R.** **95% CI (lower-upper)**
Pairsex	0.33 (0.05–2.12)	0.26 (0.02–3.97)
Race	0.44 (0.12–1.58)	0.22 (0.03–1.60)
Hispanic/Non-Hispanic	2.53 (0.24–26.31)	10.66 (0.74–154.41)
Income	0.86 (0.23–3.26)	1.02 (0.29–3.56)
Age	1.02 (0.98–1.06)	1.05 (1.00–1.10)
Education	2.98 (0.99–8.97)	6.05 (1.65–22.14)
Relationship status	0.47 (0.16–1.37)	0.38 (0.06–2.35)
Employment	0.80 (0.17–3.70)	0.77 (0.12–5.07)
Student/Non-student	0.65 (0.12–3.45)	0.98 (0.08–11.56)
